# Insect-Inspired Sequential Inspection Strategy Enables an Artificial Network of Four Neurons to Estimate Numerosity

**DOI:** 10.1016/j.isci.2018.12.009

**Published:** 2018-12-14

**Authors:** Vera Vasas, Lars Chittka

**Affiliations:** 1 School of Biological and Chemical Sciences, Queen Mary University of London, London E1 4NS, UK; 2 Wissenschaftskolleg zu Berlin, Institute for Advanced Study, Berlin 14193, Germany

**Keywords:** Neuroscience, Cognitive Neuroscience, Biocomputational Method

## Abstract

Varying levels of numerical cognition have been found in several animal species. Bees, in particular, have been argued to be able to count up to four items and solve complex numerical tasks. Here we present an exceedingly simple neural circuit that, when provided with the actual visual input that the bee is receiving while carrying out the task, can make reliable estimates on the number of items in the display. Thus we suggest that the elegance of numerical problem solving in bees might not lie in the formation of numerical concepts (such as “more,” “less,” or “zero”), but in the use of specific flight movements to scan targets, which streamlines the visual input and so renders the task of counting computationally inexpensive. Careful examination of the actual inspection strategies used by animals might reveal that animals often employ active scanning behaviors as shortcuts to simplify complex visual pattern discrimination tasks.

## Highlights

• Small-brained animals such as bees can solve counting tasks
• Exceedingly small neural circuits can mediate numerosity estimations
• The method requires a sequential inspection strategy to generate the visual input
• Active scanning behavior is suggested to play a role in complex cognitive tasks

## Graphical Abstract

**Figure:**
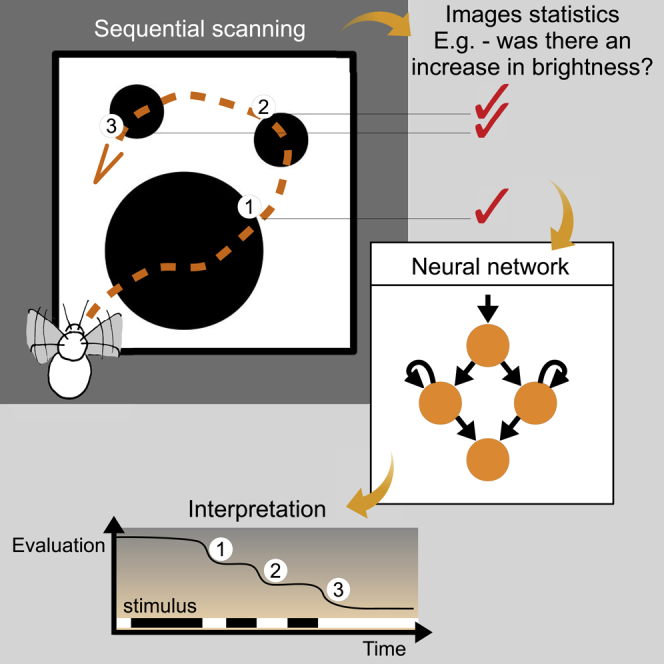

## Introduction

Numerical cognition is traditionally considered a higher cognitive ability, perhaps because of its association with the most advanced human intellectual achievements. The symbolic, language-based mathematics we use, however, appears to be rooted in a predisposition to use quantitative information without symbolic representation, which exists in pre-verbal infants and in cultures that do not use symbols for counting (Dehaene, 2001, Feigenson et al., 2004). A growing body of experiments demonstrates that a wide range of animals possess a similar “number sense.” Not only birds (Nieder, 2018, Pepperberg, 2006, Rugani, 2018) and mammals (Matsuzawa, 2009, Nieder, 2018) or other large-brained animals but also fish, frogs/toads, and even insects with miniature brains were shown to be able to make decisions based on numerosity (reviewed in Agrillo and Bisazza, 2014, Agrillo and Bisazza, 2018, Pahl et al., 2013, Rose, 2018, Skorupski et al., 2018). Bees, in particular, exhibit counting-like abilities and can be trained to search for food after a given number of landmarks (Chittka and Geiger, 1995, Dacke and Srinivasan, 2008, Menzel et al., 2010) or on the stimulus with a given number of items (Skorupski et al., 2018), and can use the number of items as the decision criteria in a match-to-sample task (Gross et al., 2009). Recently, honeybees have been argued to even understand numerical concepts of “less than,” “greater than,” and “zero” as a number (Howard et al., 2018).

However, how complex is numerical cognition in neurocomputational terms? Computer vision algorithms, often based on convolutional neural networks, are abundantly used for counting objects in images and offer a good starting point for addressing this question (e.g., Dijkstra et al., 2018 and references therein). Most of these algorithms rely on object detection and then count the detected instances, but because such methods explicitly make use of symbolic mathematics, they are not accessible for animal brains. However, computer vision has also proved that it is possible to reliably estimate object count without detecting and localizing individual object instances, using, for example, image density (Lempitsky and Zisserman, 2010, Rahnemoonfar and Sheppard, 2017). As for the size of the neural network necessary, Dehaene and Changeux (1993) proposed a formal model of only 480 neural units (plus 50 input units) to account for the elementary numerical abilities of infants and animals. This model is able to extract approximate numerosity from images, up to five items. It seems likely that the perception of numerosity is a basic attribute of visual systems (Burr and Ross, 2008) and emerges spontaneously when neural networks are trained to encode statistical properties of images (Stoianov and Zorzi, 2012).

Here we explore how serial processing reduces the size of the neural hardware required for basic counting. It seems likely that bees cannot extract complex visual pattern properties “at a glance” (Nityananda et al., 2014); they inspect pattern elements from up close (Guiraud et al., 2018, Ings et al., 2012) and one by one (Skorupski et al., 2018). If such an inspection strategy is indeed universal, it has profound implications for the complexity of visual tasks. We present a simple abstract model of only four neural units that, when provided with the responses that known low-level visual neurons would produce during such a sequential scan, is able to match the bees' performance in a complex numerical ordering task (Howard et al., 2018, Skorupski et al., 2018). The output of this network is sufficient to distinguish between numerosities up to 4–6, produces an appropriate response to an empty set (“zero”), and reproduces Weber's law of number discriminability.

## Results

**Figure 1:**
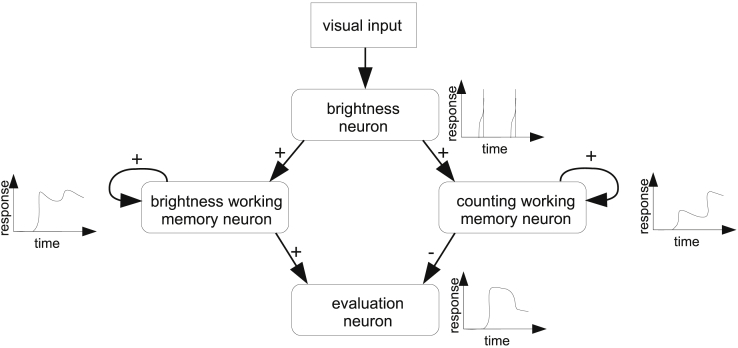
Simple Neural Model for Counting The phasic “brightness” neuron extracts the change in brightness from the visual input. The working memory neurons in the second layer are recurrent and thus maintain exponentially decaying memory traces. The “brightness working memory” neuron receives strong input from the “brightness” neuron, and signals recent changes in brightness. The “counting working memory” neuron receives weak input from the “brightness” neuron, and so accumulates information about the changes in brightness over a longer period. Finally, the “evaluation” neuron subtracts the “counting working memory” from the “brightness working memory.” Its response is inversely proportional to the number of brightness changes, and, with the right visual input, it provides an online evaluation of the numerosity of the stimulus.

Our simple model (Figure 1) employs just four independent neural units (which we will refer to as neurons for simplicity). It is able to mimic the counting abilities of bees—provided that it receives sequential visual input of the countable items. The first neuron is a wide-field (60° visual angle) neuron that sums up the responses of a collection of phasic on-off narrow-field cells of the medulla (Arenz et al., 2017). Neurons with similar response properties have been found in the second and third visual ganglia (medulla and lobula) of insects (Douglass and Strausfeld, 2003, Hertel, 1980, Paulk et al., 2009a, Yang and Maddess, 1997). This phasic “brightness” neuron responds to changes in brightness within its receptive field. The model is not limited to this specific type of input but will provide comparable results when using input from a global brightness detector or an edge detector (Figure S1). The “brightness working memory” neuron receives strong excitatory input from the “brightness” neuron and feeds back to itself; thus its response will be close to maximum when the bee encounters a change in light intensity. The “counting working memory” neuron also feeds back to itself, but it is only weakly stimulated by the “brightness” neuron; thus its response will be proportional to the number of times the bee has moved between dark and bright areas. Note that numbers are not registered as integers, but accumulated as magnitudes (as in the approximate number system described in humans for estimating numerosities higher than 4; Feigenson et al., 2004). Finally, the “evaluation” neuron is excited by the “brightness working memory” neuron and inhibited by the “counting working memory” neuron. The “evaluation” neuron thus accumulates information about the stimulus while the bee is inspecting it, and so this neuron provides a continuously updating evaluation of the numerosity of the stimulus (Figure 1).

**Figure 2:**
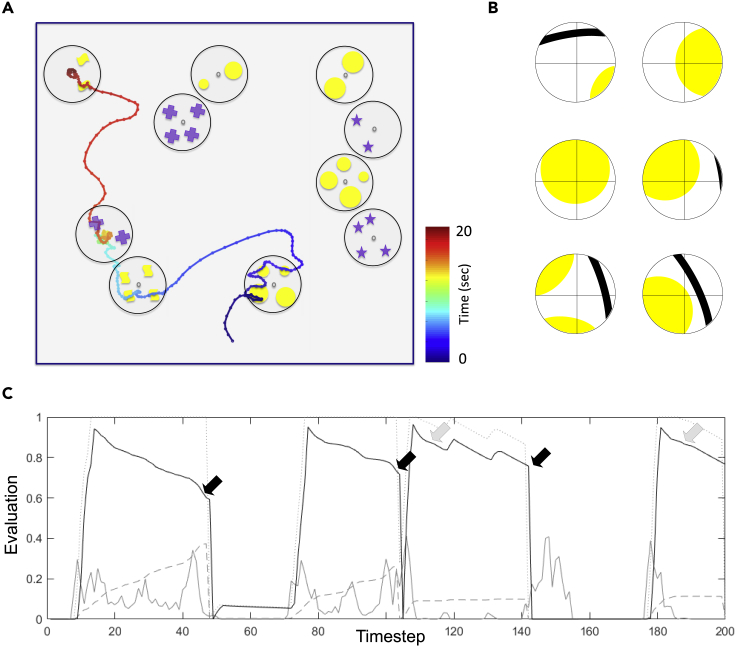
Estimating Numerosity in a Counting Task Using realistic visual input and following simple rules for interpreting the evaluation provided by the neural network, the model can provide enough information to reproduce the decisions the bee made in the counting task from Skorupski et al. (2018). (A) The flight path of a bee trained to choose two items and not four items. The bee inspects each item one by one, flying over them at a distance of 1–2 cm. During training, the bee was rewarded with sugar solution hidden in a hole in the middle of the correct stimulus (indicated by a small circle). When a stimulus is chosen, the bee hovers in front of the hole, trying to feed from it; when rejected, she leaves the stimulus without trying to feed. Reproduced from Skorupski et al. (2018). (B) Example set of visual input to the model during scanning. From a short distance, with limited field of view, the visual input is akin to moving a spotlight across the image. (C) The responses of the “brightness” neuron (gray line), “brightness working memory” neuron (dotted line), the “counting working memory” neuron (dashed line), and the “evaluation” neuron (black line) for the flight path shown in (A). Decisions to land on a stimulus (light gray arrow) are made when the scan is finished (there are no more items in sight) and the response of the “evaluation” neuron is high (above approximately 0.8 here). Decisions to leave the stimulus (black arrows) are triggered when the “evaluation” falls below a threshold (approximately 0.8 here). When the bee decides to leave a stimulus, the network is reset, and it is reactivated once the bee has left the stimulus.

The flight path of the bee, and the resulting visual input sequence, is crucial for generating a correct evaluation. A recent experiment (Skorupski et al., 2018) analyzed the bees' flight trajectories when choosing between patterns with different number of elements and concluded that bees inspect pattern elements sequentially, flying over each item once. Moreover, bees keep very close to the pattern during scanning (Guiraud et al., 2018, Ings et al., 2012). We assumed a 1–2 cm viewing distance; from this distance, our wide-field neuron's receptive field of 60° only covers 1.2–2.3 cm in diameter of the stimulus. Thus its input is akin to a moving spotlight across the pattern (Figure 2).

Our network outputs an estimate of the numerosity that can be used to solve a number of different types of quantification tasks (counting, equals to, less than, or more than) depending on how the “evaluation” neuron's response is interpreted in decision making. For the “choose two not four” task from Skorupski et al. (2018), we employed the rule that the bee is prompted to leave the stimulus if the “evaluation” neuron's response falls below a critical level (approximately 0.8 here). This can happen if the bee has passed over more items than she is looking for (>3 items here) and when she has been searching for the sugar reward without success long enough for the working memory to degrade. We also assume that the bee would land on the stimulus should she complete a scan without being prompted to abandon it. In either event, the decision to leave or find the sugar reward will reset the network by inhibiting both the “brightness working memory” and the “counting working memory” neurons. Using these assumptions, we find that the model reliably predicts the rejections and landings shown in Skorupski et al. (2018) based on the visual input during its flight path (Figure 2). The same model outputs can be used for selecting “higher number” or “greater” by inverting the decision rule, i.e., the bee should leave the stimulus after finishing the scan but lands on it once the evaluation falls below the threshold. Moreover, the same method can be used to address landmark counting (Chittka and Geiger, 1995, Dacke and Srinivasan, 2008). Here, the bee is viewing large landmarks from a distance, instead of small items from close up; the behavioral rule is to interrupt flight and land when the “evaluation” neuron's response falls below a threshold.

**Figure 3:**
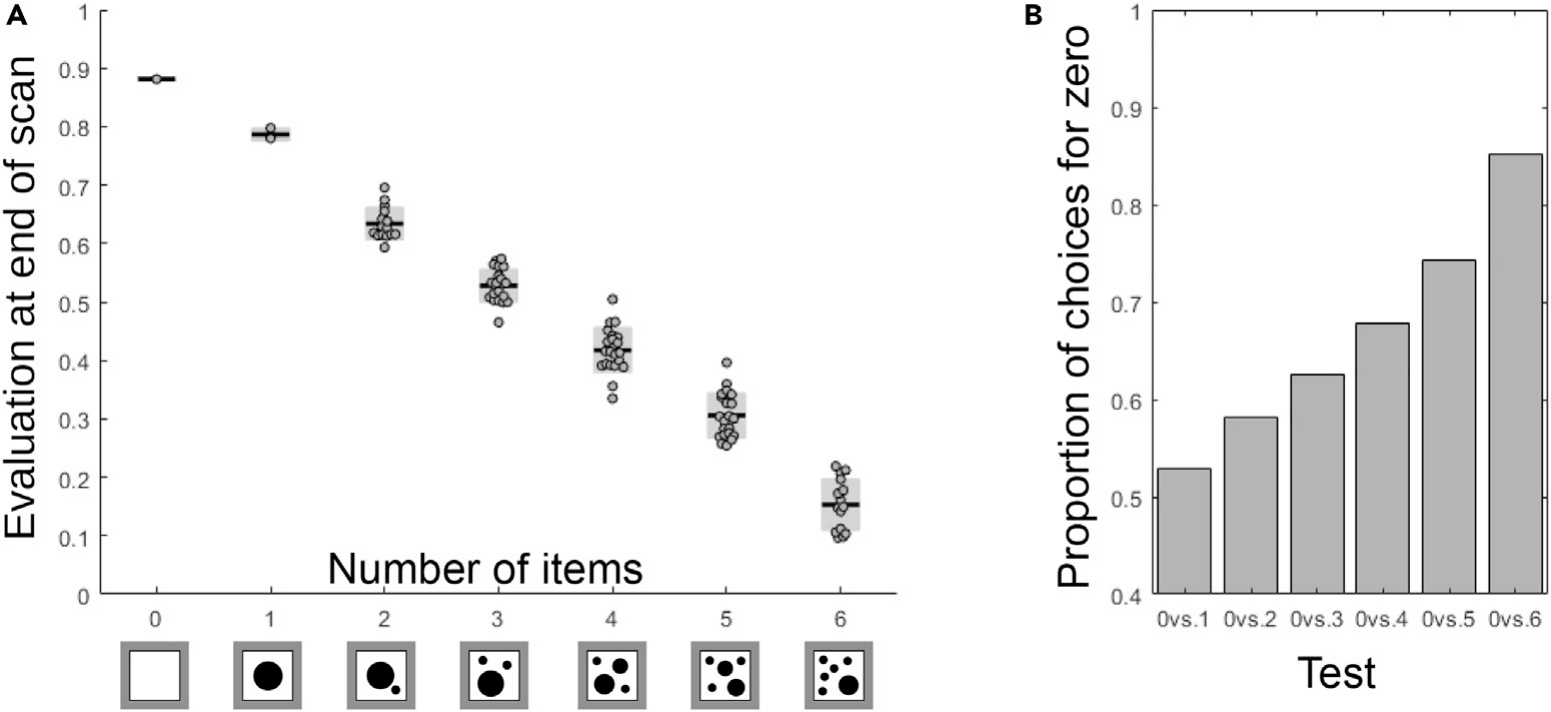
Estimating Numerosity in a Numerical Ordering Task The model reproduces the choices of the bee in a complex numerical ordering task from Howard et al. (2018), including the preference for the empty stimulus (“zero”) and an increasing success of discrimination with increasing numerical distance (A) The evaluation given by the model for the stimuli used in Howard et al. (2018) shows a decreasing response with increasing numerosity. Each stimulus contains a varying number (0–6) of differently shaped (circle, triangle, or square) items. 1, 3, 15, or 21 individual patterns per number were used. The dots represent the “evaluation” responses to each pattern, the black lines indicate their means, and the gray areas depict the standard deviations. We assumed the bee scans each pattern by flying over each item once, as described in Skorupski et al. (2018). (B) If the bee chooses patterns to scan randomly and lands with a likelihood directly proportional to the state of the “evaluation neuron” at the end of the scan, the distribution of landings on zero versus other numerosities follows Weber's law of number discriminability (as found in Howard et al., 2018). Note that this decision rule does not involve comparing two stimuli. See also Figure S1 and Table S1.

We then turned our attention to a recent study in which bees succeeded in numerical ordering tasks (including an adequate response to zero), which was interpreted by the authors as indicating that bees form concepts of “less than,” “greater than,” and “zero” (Howard et al., 2018). The flight paths were not recorded in this article, compelling us instead to assume an idealized flight path based on the scanning rules described in Skorupski et al. (2018), one that passes over each item once, at a hovering distance of 2 cm. The decision to land on the feeder is determined by the value of the “evaluation” neuron after completing the scan. For the “less than” task, the decision to land would be guided by a high value, whereas the “more than” task would be guided by a lower value. Note that the network does not compare two sets of stimuli; instead, a decision is made concerning each stimulus independently based on its overall score. We simulated the input to the neural network that could result from flying a scanning path and calculated the output from the “evaluation” neuron to predict the probability of choosing a pattern. For the stimuli used in Howard et al. (2018), the model's numerosity estimations match the performance of the bees (Figure 3 and Table S1—“less than” task; for the “more than” task, see Table S1). The model's accuracy when choosing from two stimuli follows Weber's law, which states that accuracy is expected to improve with numerical distance. Finally, the model reliably rates “zero” (an empty sheet) over other numbers in the “less than” task. Our counting network actually outperformed the behavior of real bees; they failed at choosing zero over two in the original experiment.

## Discussion

The model rests on the assumption that during counting bees scan pattern elements sequentially and visit each element once (proposed in Skorupski et al., 2018). There is evidence to show that this scanning behavior might be required to extract visual pattern information. The time bees require to solve a visual discrimination task increases with task complexity (Ings et al., 2012, Nityananda et al., 2014), indicating that they use some sort of sequential scanning behavior. As bees observe pattern elements from a distance of only a few centimeters (Guiraud et al., 2018, Ings et al., 2012), the larger part of the pattern will be out of the bees' field of view at any given time, suggesting a one-by-one inspection strategy. How bees know which pattern element they have already visited is not yet fully understood; however, as they can clearly avoid revisits to previously emptied feeders (Lihoreau et al., 2012) and previously emptied nectaries within one flower based on their position (Bar-Shai et al., 2011), we expect them to be able to use their working memory to avoid revisits to pattern elements as well. The visual inspection strategies that other non-human animals use during counting remain unexplored; there is, however, evidence for animals following a set visual path during object recognition tasks (in chickens; Dawkins and Woodington, 2000). Overall, the sequential scanning behavior described in Skorupski et al. (2018) might be a version of a more universal strategy, co-opted and tailored to the task of counting.

The model offers a non-countable magnitude estimation similar to the core system 1 humans employ for approximating large numbers, and not the core system 2 we use to discriminate a small number of items “at a glance” (subitizing) (Feigenson et al., 2004; but see Gallistel and Gelman, 2000 for the argument that both systems use magnitude estimation). As the success of discrimination is expected to be ratio dependent only in the first case, and this has been argued for bees in recent experiments (Howard et al., 2018), there is some support for this kind of implementation, but more experiments will be needed. Consider, however, that there are other known examples of approximate magnitude estimation circuits in insect brains. Ants, for example, measure distance by integrating step count (Wittlinger et al., 2006, Wittlinger et al., 2007), whereas bees keep track of the total amount of image motion in the lateral regions of their visual field (Srinivasan et al., 1997, Stone et al., 2017). The “item counter” might use the same neural architecture as these distance meters, or even recruit the visual distance circuit directly.

The crucial element in our neural architecture is the implementation of this “item counter” as a recurrent working memory circuit. Similar configurations have been found in insect brains (Douglass and Strausfeld, 2003, Eichler et al., 2017, Grünewald, 1999, Haag and Borst, 2001), and recurrent microcircuits have been suggested to serve the role of memory units encoding the distance traveled during path integration (Stone et al., 2017). In our model, recurrence creates an exponentially decaying memory trace that can be modified by further inputs. Behavioral experiments using a delayed-matching-to-sample paradigm have established that the bees' working memory (early short-term memory) indeed degrades in an exponential fashion and disappears in less than 10 s (Raine and Chittka, 2011, Zhang et al., 2005). In our model, we used one neuron (as theorized in Loewenstein and Sompolinsky, 2003). However, working memory is more likely to be implemented by a group of recurrently wired neurons. The memory capacity of such recurrent neural networks, called echo-state networks, depends on their size and on the level of noise (Jaeger, 2002); a small network, receiving the inevitably noisy visual input, is in line with the limited counting abilities of bees and of many other species.

The location of visual working memory in the bee brain is unknown. For olfactory learning, the antennal lobes have been identified as most likely sites for working memory/early short-term memory (Erber et al., 1980, Menzel, 1999); the most likely analog sites in the visual pathway would be in the medulla or lobula. In the medulla, recurrently wired neurons might exist in the serpentine layer (Douglass and Strausfeld, 2003); in the lobula, in the inner layers (Haag and Borst, 2001). There is evidence that in flies some short-term memory traces are stored in the central complex (Liu et al., 2006); for bees, Stone et al. (2017) put forward an argument for a recurrent microcircuit serving as the memory unit in the path integrator in the central complex. The linear neurons we used here are capable of less computation than a more complex spiking neuron, and even if we need to scale up the number of working memory neurons an order of magnitude to counterbalance neural noise, the necessary tens of neurons would still fit easily in the optic lobes or the central complex. Two simple working memory circuits, each of which receives the same input only weighted differently, may be easily co-opted for counting by neurons in the mushroom body (Eichler et al., 2017, Grünewald, 1999; represented in our model as one “evaluation” neuron). To summarize, neurons of the types that are required for our simple theoretical model do exist in the insect brain. Although it is likely that more than four neurons are involved in real counting tasks (perhaps with multiple similar circuits operating in parallel), such circuitry would still not be prohibitively expensive to be accommodated in an insect brain.

The advantage of explicitly outlining a possible neural architecture is that such a model yields testable predictions. Our model makes the following predictions (among others). (1) The counting circuit we proposed relies on inputs from the color blind motion pathway, and so expected to use only long-wavelength-sensitive receptor inputs and be color blind as well (Paulk et al., 2008, Paulk et al., 2009b). (2) The signal of the working memory circuit that acts as an “item counter” degrades exponentially with time; thus introducing delays during a scan is expected to cause the bee to underestimate numerosity. (3) We propose that the bee tailors her scan to the stimulus, in a way that keeps input noise to the minimum; forcing the bee to an altered scan route (e.g., viewing the stimulus from a larger distance than ideal) should degrade the performance. (4) The model makes the decision to land on a stimulus based on its numerosity, without directly comparing two stimuli. With appropriate tuning, this decision rule is enough to reliably choose the smaller or the larger numerosity but cannot be used to choose the middle number from three numbers; thus bees should fail at his task.

Fifty years ago, the ability to learn concepts was considered uniquely human and a sign of the highest form of intelligence. We now know from behavioral experiments that bees *can* solve the delayed-match-to-sample (Gross et al., 2009), sameness/difference (Giurfa et al., 2001), and above/below tasks (Avarguès-Weber et al., 2011), and can count and extrapolate to zero (Howard et al., 2018), but the behavioral strategies by which they do so and whether these indeed require the formation of concepts is a separate question. Our artificial neural network of a few nodes, when given appropriately structured visual input, can solve a variety of tasks without requiring any form of concept or “understanding.” The model demonstrates this principle applied to counting and numerical ordering; however, counting is not the only learning task whose computational complexity has been called into question recently. After viewing an image, bees are able to distinguish between the same and a novel image, and can be trained to choose either the same or the different one, a task that appears to require the concepts of sameness and difference (Giurfa et al., 2001); modeling work, however, suggested a simple but neurobiologically plausible circuit that matches the bees' performances, and which does not involve any top-down processing (Cope et al., 2018). In the “above-and-below” spatial conceptual task, bees are required to decide if any object is above or below a referent (Avarguès-Weber et al., 2011). A recent behavioral study that examined the bees' flight paths during this task (Guiraud et al., 2018) proposed that bees could turn the spatial relation task into a simple discrimination task by only inspecting the bottom part of any pattern and making a decision based on whether it is the referent (above which the item in question is found, thus the answer is “above”) or anything else (thus the answer is “below”). Honeybees have been shown to selectively opt out from making a choice when they are uncertain, an indication of metacognition; however, the authors argue that the same neural circuit that implements simple associative learning could govern the behavior (Perry and Barron, 2013). Although it is often argued that cognitive ability correlates with brain size (see, e.g., Kotrschal et al., 2013 for an example on numerical abilities in guppies), our results and these studies show that seemingly advanced cognitive performance can be achieved with extremely small circuits. If this is so, a careful re-examination of the potential evolutionary advantage of bigger brains is in order (Chittka and Niven, 2009).

In comparative cognition, there is little value in rating cognitive task difficulty based on how difficult it is using symbolic human-like thinking. What is worthy of our attention is the repertoire of innate and learnt behavioral routines that animals employ while completing the task, and the complexity of the task in terms of neurocomputation. Within this framework we have shown that counting and numerical ordering are computationally inexpensive, provided the animal employs an active, sequential scanning of pattern elements. Here we studied the scanning behavior of bees; similarly simple computational solutions may underpin numerical cognition in other animals that employ active scanning (e.g., Chittka and Skorupski, 2017, Dawkins and Woodington, 2000, Gegenfurtner, 2016). Furthermore, we have shown that counting does not need to rely on the internal representation of concepts. Sequential scanning drastically reduces the demand for the neural hardware required to solve the task. We conclude that active scanning behavior could play a major role in even the most complex cognitive tasks. Future studies in comparative cognition should benefit from shifting the focus from *what* an animal can do to *how* it does it and explore the intricacies of the sequential decision-making process (Chittka et al., 2012).

### Limitations of the Study

(1) The model parameters were chosen to demonstrate that the circuit is able to estimate numerosity, but we do not investigate the learning or other processes that lead to the emergence of the synaptic weight parameters. (2) We do not test the robustness of the model to variations in input stimuli other than that inherent in the stimuli used in Skorupski et al. (2018) and Howard et al. (2018). We decided to omit these tests as a realistic estimate of model robustness would require more information on the scanning behavior (and thus the visual input) and the neural implementation than available.

## Methods

All methods can be found in the accompanying Transparent Methods supplemental file.

## Supplemental Information

Document S1. Transparent Methods, Figures S1 and S2, and Table S1

Data S1. Supplemental Script Files, Related to Methods and Figures 1–3 Custom MATLAB scripts for modeling the minimal neural network, and the associated input files: the stimuli and the flight paths from Howard et al. (2018) and Skorupski et al. (2018).

## References

[bib1] Agrillo C., Bisazza A. (2018). Understanding the origin of number sense: a review of fish studies. Philos. Trans. R. Soc. Lond. B Biol. Sci..

[bib2] Agrillo C., Bisazza A. (2014). Spontaneous versus trained numerical abilities. A comparison between the two main tools to study numerical competence in non-human animals. J. Neurosci. Methods.

[bib3] Arenz A., Drews M.S., Richter F.G., Ammer G., Borst A. (2017). The temporal tuning of the *Drosophila* motion detectors is determined by the dynamics of their input elements. Curr. Biol..

[bib4] Avarguès-Weber A., Dyer A.G., Giurfa M. (2011). Conceptualization of above and below relationships by an insect. Proc. Biol. Sci..

[bib5] Bar-Shai N., Keasar T., Shmida A. (2011). The use of numerical information by bees in foraging tasks. Behav. Ecol..

[bib6] Burr D., Ross J. (2008). A visual sense of number. Curr. Biol..

[bib7] Chittka L., Geiger K. (1995). Can honey bees count landmarks?. Anim. Behav..

[bib8] Chittka L., Niven J. (2009). Are bigger brains better?. Curr. Biol..

[bib9] Chittka L., Rossiter S.J., Skorupski P., Fernando C. (2012). What is comparable in comparative cognition?. Philos. Trans. R. Soc. Lond. B Biol. Sci..

[bib10] Chittka L., Skorupski P. (2017). Active vision: a broader comparative perspective is needed. Constr. Found.

[bib11] Cope A.J., Vasilaki E., Minors D., Sabo C., Marshall J.A.R., Barron A.B. (2018). Abstract concept learning in a simple neural network inspired by the insect brain. PLoS Comput. Biol..

[bib12] Dacke M., Srinivasan M.V. (2008). Evidence for counting in insects. Anim. Cogn..

[bib13] Dawkins M.S., Woodington A. (2000). Pattern recognition and active vision in chickens. Nature.

[bib14] Dehaene S. (2001). Précis of the number sense. Mind Lang..

[bib15] Dehaene S., Changeux J.-P. (1993). Development of elementary numerical abilities: a neuronal model. J. Cogn. Neurosci..

[bib16] Dijkstra, K., van de Loosdrecht, J., Schomaker, L.R.B., and Wiering, M.A. (2018). CentroidNet: A deep neural network for joint object localization and counting. Conference: The European Conference on Machine Learning and Principles and Practice of Knowledge Discovery in Databases.

[bib17] Douglass J.K., Strausfeld N.J. (2003). Retinotopic pathways providing motion-selective information to the lobula from peripheral elementary motion-detecting circuits. J. Comp. Neurol..

[bib18] Eichler K., Li F., Litwin-Kumar A., Park Y., Andrade I., Schneider-Mizell C.M., Saumweber T., Huser A., Eschbach C., Gerber B. (2017). The complete connectome of a learning and memory centre in an insect brain. Nature.

[bib19] Erber J., Masuhr T., Menzel R. (1980). Localization of short-term memory in the brain of the bee, *Apis mellifera*. Physiol. Entomol..

[bib20] Feigenson L., Dehaene S., Spelke E. (2004). Core systems of number. Trends Cogn. Sci..

[bib21] Gallistel C.R., Gelman R. (2000). Non-verbal numerical cognition: from reals to integers. Trends Cogn. Sci..

[bib22] Gegenfurtner K.R. (2016). The interaction between vision and eye movements. Perception.

[bib23] Giurfa M., Zhang S., Jenett A., Menzel R., Srinivasan M.V. (2001). The concepts of “sameness” and “difference” in an insect. Nature.

[bib24] Gross H.J., Pahl M., Si A., Zhu H., Tautz J., Zhang S. (2009). Number-based visual generalisation in the honeybee. PLoS One.

[bib25] Grünewald B. (1999). Morphology of feedback neurons in the mushroom body of the honeybee, *Apis mellifera*. J. Comp. Neurol..

[bib26] Guiraud M., Roper M., Chittka L. (2018). High-speed videography reveals how honeybees can turn a spatial concept learning task into a simple discrimination task by stereotyped flight movements and sequential inspection of pattern elements. Front. Psychol..

[bib27] Haag J., Borst A. (2001). Recurrent network interactions underlying flow-field selectivity of visual interneurons. J. Neurosci..

[bib28] Hertel H. (1980). Chromatic properties of identified interneurons in the optic lobes of the bee. J. Comp. Physiol..

[bib29] Howard S.R., Avarguès-Weber A., Garcia J.E., Greentree A.D., Dyer A.G. (2018). Numerical ordering of zero in honey bees. Science.

[bib30] Ings T.C., Wang M.-Y., Chittka L. (2012). Colour-independent shape recognition of cryptic predators by bumblebees. Behav. Ecol. Sociobiol..

[bib31] Jaeger H. (2002). Short Term Memory in Echo State Networks.

[bib32] Kotrschal A., Rogell B., Bundsen A., Svensson B., Zajitschek S., Brännström I., Immler S., Maklakov A.A., Kolm N. (2013). Artificial selection on relative brain size in the guppy reveals costs and benefits of evolving a larger brain. Curr. Biol..

[bib33] Lempitsky, V. and Zisserman, A. (2010). Learning to count objects in images. NIPS10 Proc. 23rd Int. Conf. Neural Inf. Process. Syst. 1, 1324–1332.

[bib34] Lihoreau M., Raine N.E., Reynolds A.M., Stelzer R.J., Lim K.S., Smith A.D., Osborne J.L., Chittka L. (2012). Radar tracking and motion-sensitive cameras on flowers reveal the development of pollinator multi-destination routes over large spatial scales. PLoS Biol..

[bib35] Liu G., Seiler H., Wen A., Zars T., Ito K., Wolf R., Heisenberg M., Liu L. (2006). Distinct memory traces for two visual features in the *Drosophila* brain. Nature.

[bib36] Loewenstein Y., Sompolinsky H. (2003). Temporal integration by calcium dynamics in a model neuron. Nat. Neurosci..

[bib37] Matsuzawa T. (2009). Symbolic representation of number in chimpanzees. Curr. Opin. Neurobiol..

[bib38] Menzel R. (1999). Memory dynamics in the honeybee. J. Comp. Physiol. A.

[bib39] Menzel R., Fuchs J., Nadler L., Weiss B., Kumbischinski N., Adebiyi D., Hartfil S., Greggers U. (2010). Dominance of the odometer over serial landmark learning in honeybee navigation. Naturwissenschaften.

[bib40] Nieder A. (2018). Evolution of cognitive and neural solutions enabling numerosity judgements: lessons from primates and corvids. Philos. Trans. R. Soc. Lond. B Biol. Sci..

[bib41] Nityananda V., Skorupski P., Chittka L. (2014). Can bees see at a glance?. J. Exp. Biol..

[bib42] Pahl M., Si A., Zhang S. (2013). Numerical cognition in bees and other insects. Front. Psychol..

[bib43] Paulk A.C., Dacks A.M., Gronenberg W. (2009). Color processing in the medulla of the bumblebee (Apidae: *Bombus impatiens*). J. Comp. Neurol..

[bib44] Paulk A.C., Dacks A.M., Phillips-Portillo J., Fellous J.-M., Gronenberg W. (2009). Visual processing in the central bee brain. J. Neurosci..

[bib45] Paulk A.C., Phillips-Portillo J., Dacks A.M., Fellous J.-M., Gronenberg W. (2008). The processing of color, motion, and stimulus timing are anatomically segregated in the bumblebee brain. J. Neurosci..

[bib46] Pepperberg I.M. (2006). Grey parrot numerical competence: a review. Anim. Cogn..

[bib47] Perry C.J., Barron A.B. (2013). Honey bees selectively avoid difficult choices. Proc. Natl. Acad. Sci. U S A.

[bib48] Rahnemoonfar M., Sheppard C. (2017). Deep count: fruit counting based on deep simulated learning. Sensors.

[bib49] Raine N.E., Chittka L. (2011). Flower constancy and memory dynamics in bumblebees (Hymenoptera: Apidae: *Bombus*). Entomol. Gen..

[bib50] Rose G.J. (2018). The numerical abilities of anurans and their neural correlates: insights from neuroethological studies of acoustic communication. Philos. Trans. R. Soc. Lond. B Biol. Sci..

[bib51] Rugani R. (2018). Towards numerical cognition’s origin: insights from day-old domestic chicks. Philos. Trans. R. Soc. Lond. B Biol. Sci..

[bib52] Skorupski P., MaBouDi H., Galpayage Dona H.S., Chittka L. (2018). Counting insects. Philos. Trans. R. Soc. Lond. B Biol. Sci..

[bib53] Srinivasan M., Zhang S., Bidwell N. (1997). Visually mediated odometry in honeybees. J. Exp. Biol..

[bib54] Stoianov I., Zorzi M. (2012). Emergence of a “visual number sense” in hierarchical generative models. Nat. Neurosci..

[bib55] Stone T., Webb B., Adden A., Weddig N.B., Honkanen A., Templin R., Wcislo W., Scimeca L., Warrant E., Heinze S. (2017). An anatomically constrained model for path integration in the bee brain. Curr. Biol..

[bib56] Wittlinger M., Wehner R., Wolf H. (2007). The desert ant odometer: a stride integrator that accounts for stride length and walking speed. J. Exp. Biol..

[bib57] Wittlinger M., Wehner R., Wolf H. (2006). The ant odometer: stepping on stilts and stumps. Science.

[bib58] Yang E.-C., Maddess T. (1997). Orientation-sensitive neurons in the brain of the honey bee (*Apis mellifera*). J. Insect Physiol..

[bib59] Zhang S., Bock F., Si A., Tautz J., Srinivasan M.V. (2005). Visual working memory in decision making by honey bees. Proc. Natl. Acad. Sci. U S A.

