# Supplementary material for: Insect-Inspired Sequential Inspection Strategy Enables an Artificial Network of Four Neurons to Estimate Numerosity
Source: iScience. 2018 Dec 14;11:85–92. doi: 10.1016/j.isci.2018.12.009 (PMC6308245; doi:10.1016/j.isci.2018.12.009)
Supplement: Document S1. Transparent Methods, Figures S1 and S2, and Table S1 [file mmc1.pdf]

ISCI, Volume *11*

Supplemental Information

Insect-Inspired Sequential Inspection Strategy Enables an Artificial Network of Four Neurons to Estimate Numerosity

Vera Vasas and Lars Chittka

Transparent Methods

CONTACT FOR REAGENT AND RESOURCE SHARING

Further information and requests for resources and reagents should be directed to and will be fulfilled by the Lead Contact, Vera Vasas (v.vasas@qmul.ac.uk or vvasas@gmail.com).

METHOD DETAILS

Generating the visual input

The relative amount of light (quantum catch) absorbed by the bee eye was set to 1 for white areas, 0 for black areas, and 0.5 for grey background areas. For the yellow and purple shown on Figure 2, the quantum catch for each receptor type can be calculated as (Backhaus and Menzel, 1987; Chittka and Kevan, 2005)

$$P = \int_{300}^{700} I_S(\lambda) S(\lambda) D(\lambda) d\lambda$$

where $I_S(\lambda)$ is the spectral reflectance function of the stimulus (Figure S2), $S(\lambda)$ is the spectral sensitivity function of the receptors from Peitsch et al. (1992), and $D(\lambda)$ is the spectrum of illumination, assumed to be unit across the visible spectrum but missing UV (matching experimental details for Skorupski et al., 2018). We used the response of the long-wavelength-sensitive receptors as the total quantum catch. (Colour and motion are processed separately in the bee brain (Paulk et al., 2008), and the latter only receives input from long-wavelength-sensitive receptors. Our model assumes input from small field on-off neurons of the motion pathway (as in e.g. Arenz et al., 2017). The quantum catch was scaled to yield 1 for white.

To calculate the visual input at each time step, we used a visual angle of 60 degrees and a viewing distance of 2 cm. We generated a partial image representing the field of view centred on a point on the flight path. The path used was the track specified on Figure 2 for the counting task (Skorupski et al., 2018) and idealized tracks (Data S1) for the numerical ordering task (Howard et al., 2018). Finally, the ‘brightness’ neuron’s responses at each time step were calculated as taking the absolute differences between the quantum catches at the current and

the previous position pixel-by-pixel, and summing them, mimicking a summation of a collection of small field on-off visual neurons.

Neural model

In our minimal model, we used abstract linear transfer neurons, whose responses are bounded by 0 and 1. Thus, the output rates of neurons r_i at time *t* are given by the following piecewise linear transfer function:

if $x_i^t < 0$, $r_i^t = 0$;

if $0 \le x_i^t \le 1$, $r_i^t = x_i^t$;

if $1 < x_i^t$ $r_i^t = 1$;

where x_i^t, the presynaptic input to neuron *i* at time *t* is calculated as the linear summation of input from presynaptic neurons *j*:

$$x_i^t = \sum_j w_{ji} r_j^{t-1}$$

where w_{ji} is the weight of the synapse from neuron *j* to *i*.

We used the following weight parameters for modelling the counting task from Skorupski et al. (2018) and the numerical ordering from Howard et al. (2018) (i – 'brightness' input neuron, b – 'brightness working memory' neuron, c – 'counting working memory' neuron, e - 'evaluation' neuron): $w_{ib} = 1.2$; $w_{ic} = 0.075$; $w_{bb} = 0.99$; $w_{cc} = 0.999$; $w_{be} = 1$; $w_{ce} = -1.1$.

DATA AND SOFTWARE AVAILABILITY

Description: Data S1

We implemented the calculations in MATLAB (Release 2018A, The MathWorks, Inc., Natick, Massachusetts, United States). Data S1 includes the scripts, the example stimuli and the flight paths from Howard et al. (2018) and Skorupski et al. (2018). Instructions for using the scripts are given as comments within each script.

KEY RESOURCES TABLE

REAGENT or RESOURCE	SOURCE	IDENTIFIER
Software and Algorithms		
MATLAB	The MathWorks, Inc.	R2018A
Custom scripts for modelling the minimal neural network, and the associated input files: the stimuli and the flight paths from Howard et al. (2018) and Skorupski et al. (2018)	This paper	Data S1

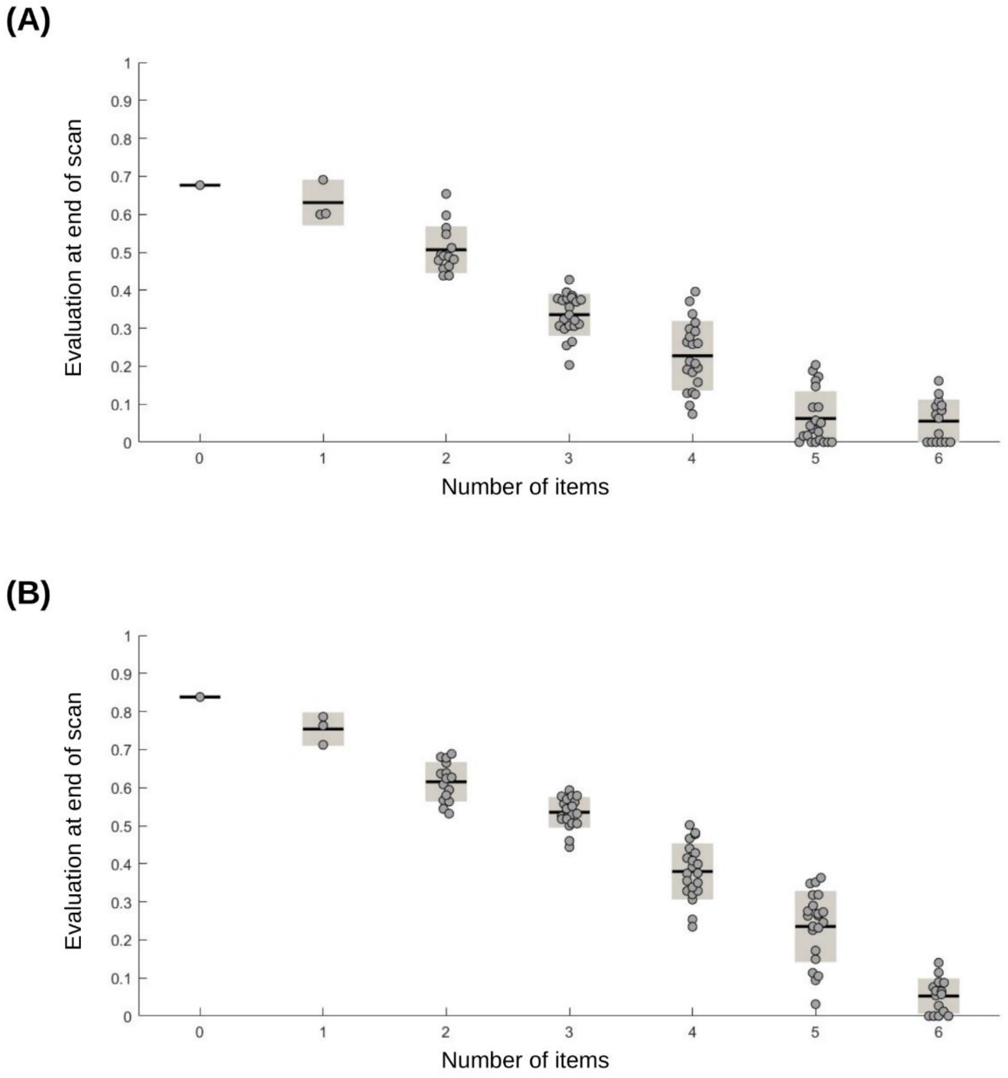

Figure S1. *The evaluation provided by the model follows the same pattern, that shown in Figure 3A, when the input is either a global brightness detector or an edge detector. Related to Figure 3.* **(A)** We calculated the global brightness neurons' response using the total amount of quantum catch from its 60-degree visual field, subtracting the value at the previous time step from that at the given time step. The result was scaled log-linearly. The global brightness detector will thus respond to an increase in brightness with a phasic burst that scales log-linearly with the extent of the change (Hertel, 1980). We calculated the evaluation given by the model for the stimuli from Howard et al. (2018) using the following parameters: $w_{ib} = 0.8; w_{ic} = 0.09; w_{bb} = 0.99; w_{cc} = 0.999; w_{be} = 1; w_{ce} = -1.1$. **(B)** We defined the response for the edge detector neurons as the fraction of its 60-degree visual field that contains edges (Yang and Maddess, 1997). We calculated the evaluation given by the model for the stimuli from Skorupski et al. (2018) using the following parameters: $w_{ib} = 1.5; w_{ic} = 0.12; w_{bb} = 0.99; w_{cc} = 0.999; w_{be} = 1; w_{ce} = -1.1$. The dots represent the response levels from the 'evaluation' neuron for each pattern; the black lines and the grey areas indicate the means and the standard deviations respectively.

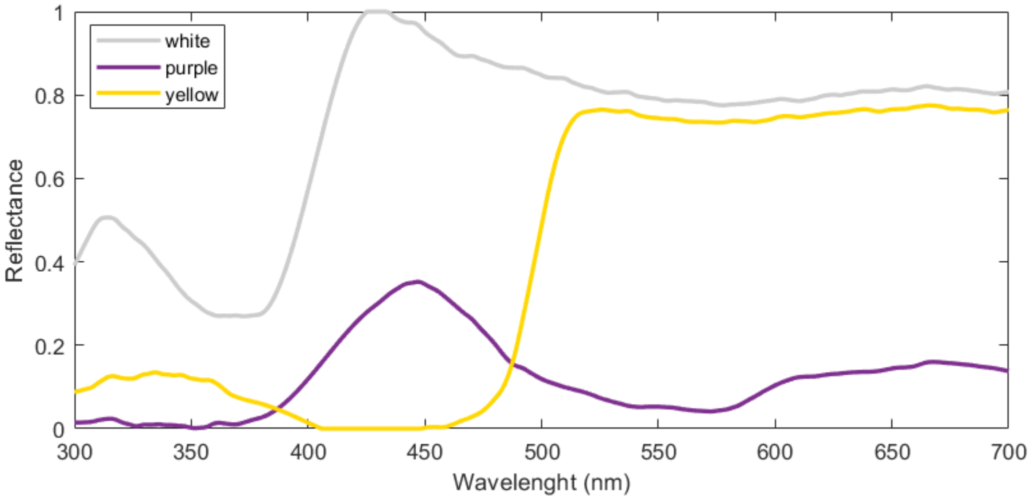

Figure S2. *The spectral reflectance functions of the colours used in Skorupski et al. (2018). Related to Figure 2 and Methods.*

A.

vs.	*0 items*	*1 item*	*2 items*	*3 items*	*4 items*	*5 items*	*6 items*
0 item	0.5	0.53	0.58	**0.63**	**0.68**	**0.74**	**0.85**
1 item	0.47	0.5	0.55	**0.6**	**0.65**	**0.72**	**0.84**
2 items	0.42	0.45	0.5	0.55	**0.6**	**0.67**	**0.81**
3 items	0.37	0.4	0.45	0.5	0.56	**0.63**	**0.77**
4 items	0.32	0.35	0.4	0.44	0.5	0.58	**0.73**
5 items	0.26	0.28	0.33	0.37	0.42	0.5	**0.67**
6 items	0.15	0.16	0.19	0.23	0.27	0.33	0.5

B.

vs.	*0 items*	*1 item*	*2 items*	*3 items*	*4 items*	*5 items*	*6 items*
0 item	0.5	0.36	0.24	0.2	0.17	0.15	0.12
1 item	**0.64**	0.5	0.37	0.31	0.27	0.24	0.2
2 items	**0.76**	**0.63**	0.5	0.44	0.39	0.35	0.3
3 items	**0.8**	**0.69**	0.56	0.5	0.45	0.4	0.36
4 items	**0.83**	**0.73**	**0.61**	0.55	0.5	0.46	0.41
5 items	**0.85**	**0.76**	**0.65**	**0.6**	0.54	0.5	0.45
6 items	**0.88**	**0.8**	**0.7**	**0.64**	0.59	0.55	0.5

Table S1. *The same model is capable of selecting 'less' or 'more' depending on the decision rule. Related to Figure 3.* The numbers indicate the proportion of landings on the stimulus indicated in the rows vs. the stimulus in the columns. **Bold** letters highlight a preference ≥ 60%. **(A)** If the bee chooses patterns to scan randomly and lands with a likelihood directly proportional to the state of the 'evaluation neuron' at the end of the scan, then the bee will show a measureable preference for the stimulus containing fewer items. **(B)** If the bee chooses patterns to scan randomly, but lands with a likelihood inversely proportional to the state of the 'evaluation neuron' at the end of the scan, then the bee will show a measureable preference for the stimulus containing more items.

Supplemental References

Arenz, A., Drews, M.S., Richter, F.G., Ammer, G., and Borst, A. (2017). The temporal tuning of the *Drosophila* motion detectors is determined by the dynamics of their input elements. Curr. Biol. 27, 929–944.

Backhaus, W., and Menzel, R. (1987). Color distance derived from a receptor model of color vision in the honeybee. Biol. Cybern. 55, 321–331.

Chittka, L., and Kevan, P.G. (2005). Flower colour as advertisement. In Practical Pollination Biology, Dafni, A., Kevan, P.G., Husband, B.C.,eds. (Enviroquest Ltd.), pp. 157-196.

Hertel, H. (1980). Chromatic properties of identified interneurons in the optic lobes of the bee. J. Comp. Physiol. 137, 215–231.

Howard, S.R., Avarguès-Weber, A., Garcia, J.E., Greentree, and A.D., Dyer, A.G. (2018). Numerical ordering of zero in honey bees. Science 360, 1124–1126.

Paulk, A.C., Phillips-Portillo, J., Dacks, A.M., Fellous, J.-M., and Gronenberg, W. (2008). The processing of color, motion, and stimulus timing are anatomically segregated in the bumblebee brain. J. Neurosci. 28, 6319–6332

Peitsch, D., Fietz, A., Hertel, H., de Souza, J., Ventura, D.F., and Menzel, R. (1992). The spectral input systems of hymenopteran insects and their receptor-based colour vision. J. Comp. Physiol. A 170, 23–40.

Skorupski, P., MaBouDi, H., Galpayage Dona, H.S., and Chittka, L. (2018). Counting insects. Phil. Trans. R. Soc. B 373, 20160513.

Yang, E.-C., and Maddess, T. (1997). Orientation-sensitive neurons in the brain of the honey bee (*Apis mellifera*). J. Insect Physiol. 43, 329–336.
